# Comparative effectiveness of strategies to prevent weight gain among women with and at risk for breast cancer: a systematic review

**DOI:** 10.1186/2193-1801-2-277

**Published:** 2013-06-26

**Authors:** Zoobia W Chaudhry, Rochelle V Brown, Oluwakemi A Fawole, Renee Wilson, Kimberly A Gudzune, Nisa M Maruthur, Jodi Segal, Susan M Hutfless

**Affiliations:** Department of Medicine, Division of General Internal Medicine, Johns Hopkins University, Baltimore, MD USA; Department of Medicine, Division of Gastroenterology and Hepatology, Johns Hopkins University, Baltimore, MD USA; Welch Center for Prevention, Epidemiology, and Clinical Research, Johns Hopkins University, Baltimore, MD USA; Department of Epidemiology, Johns Hopkins University, Baltimore, MD USA; Department of Health Policy and Management, Johns Hopkins Bloomberg School of Public Health, Baltimore, MD USA; University Health Services, School of Medicine, Johns Hopkins University, 933 N. Wolfe Street, Baltimore, MD 21205 USA

## Abstract

**Background:**

Obesity affects cancer risk and treatment outcomes. Preventing weight gain may prevent some cancers, improve cancer outcomes, reduce cancer recurrence and increase cancer-related survival. We performed a systematic review to identify strategies to prevent weight gain in individuals with or at risk for breast cancer.

**Findings:**

We included 2 studies from 27,879 citations. In premenopausal women at risk for breast cancer, a low fat diet prevented weight gain at 12 months. Among women with breast cancer, effective strategies to prevent weight gain included low-fat dietary counseling with self-management techniques. One trial reported on cancer outcomes, mortality and adverse events. Low-fat dietary counseling wilth self-management techniques lowers the risk breast cancer relapse by 24% compared with less intensive counseling with maintenance of nutritional status goal. There was no difference in overall mortality and no adverse events were observed.

**Conclusion:**

Limited evidence suggests that women with or at risk for breast cancer may successfully employ dietary and exercise strategies to prevent weight gain for at least one year. Low fat dietary counseling may improve cancer outcomes in women with breast cancer. Future studies should confirm these findings and evaluate the impact of weight gain prevention on cancer incidence, recurrence and survival.

**Electronic supplementary material:**

The online version of this article (doi:10.1186/2193-1801-2-277) contains supplementary material, which is available to authorized users.

## Introduction

Overweight and obesity have been linked with increased risk of death from certain cancers such as breast cancer, renal cell carcinoma, pancreatic cancer, colon cancer and gynecologic cancers (Flegal et al. 2007). However, weight is one of the modifiable risk factors for cancer. Preventing weight gain could be an effective strategy to decrease the risk of malignancy in populations most at risk (Ballard-Barbash et al. 2012).

Cancer survivors with obesity have poorer cancer-related outcomes than healthy weight patients including a higher number of second primary cancers and (Flegal et al. 2007; Daling et al. 2009) higher cancer recurrence (Flegal et al. 2007; Ewertz et al. 2011), cancer related mortality and overall mortality rates (Flegal et al. 2007; Sinicrope et al. 2010; Ewertz et al. 2011; Protani et al. 2010). Overweight and obese individuals with cancer are also at risk of worse shortterm outcomes like poor wound healing, increased rates of postoperative infections (Albino et al. 2009; Doyle et al. 2010), and poor response to treatment (Bastarrachea et al. 1994). Overweight or obesity also increases the risk of other chronic conditions like cardiovascular disease and diabetes, that can influence cancer survivorship (Yancik et al. 2001). A recent meta-analysis found increased overall and cancer-specific mortality in obese breast cancer survivors (Protani et al. 2010). Interventions targeting the prevention of weight gain after cancer diagnosis and treatment could improve these outcomes and decrease recurrence.

Despite the theoretical benefits of weight gain prevention among populations with and at risk for breast cancer, we know of no prior synthesis of the weight gain prevention literature among this population. We aimed to review strategies to prevent weight gain in individuals at risk of breast cancer or with breast cancer. The strategies of interest were self-management, diet, physical activity, or combinations of these strategies.

## Methods

### Literature search strategy

We searched the following databases for primary studies: MEDLINE^®^, Embase, the Cochrane Central Register of Controlled Trials, CINAHL and PsycINFO through June 2012. We developed and followed a standard protocol for this review following the *Methods Guide for Effectiveness and Comparative Effectiveness Reviews* (Owens et al. 2010). Additional details of the protocol are available in our full evidence report (Hutfless et al.). Title, abstract and full article reviews were performed by two independent reviewers. Conflicts were resolved by consensus adjudication. Relevant data were extracted from eligible intervention trials. Each article was serially abstracted by a first reviewer and then by a senior reviewer. This manuscript is a subset of studies from a report on strategies to prevent weight gain. The cancer section of the report included all study designs and all cancers, although the majority of evidence was for breast cancer.

Weight outcomes of interest were body mass index (BMI), weight, and waist circumference. Cancer-related outcomes included cancer incidence, recurrence and mortality. We also sought quality of life, adherence, and safety outcomes. Safety outcomes included nutritional deficiencies, eating disorders, and activity-related injury. The data abstraction time points of interest for weight-related outcomes were 1 year, 2 years, 5 years and the last reported time point after five years of follow up. For the other outcomes, we abstracted data for the last reported time point on or after one year. This study did not qualify as human-subjects research and, therefore, was not needed to be reviewed by the IRB.

### Quality assessment of individual studies

Study quality was assessed using the Downs and Black methodologic quality assessment checklist (Downs and Black 1998). We used information on study quality to assess the risk of bias (using the internal validity items) of the studies. Two reviewers independently completed the checklist for each article and came to consensus for each item.

### Data synthesis

We examined the study designs for qualitative similarities, and noted significant heterogeneity. Therefore, studies were not amenable to pooling with meta-analyses, and we instead calculated and displayed the between group mean differences with 95 percent confidence intervals (CI) for the individual studies, when provided.

We selected meaningful between-group difference thresholds as follows: 0.5 kg for weight, 0.2 units for BMI (based on a 0.5 kg change for an individual with a BMI of 27), and 1 cm for waist circumference. The meaningful threshold was annualized to account for the different durations of the studies. A P value <0.05 was considered statistically significant. For a strategy to be considered effective, it had to meet the meaningful between group difference and statistical thresholds.

## Results

**Figure 1:**
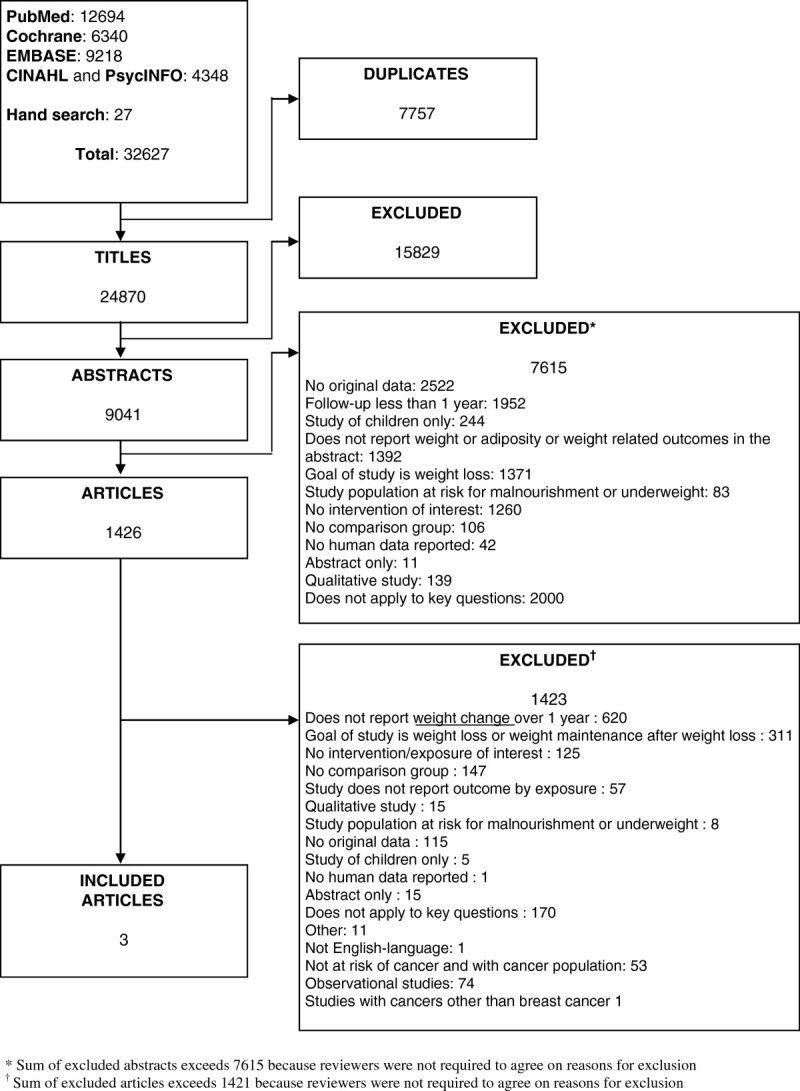
**Results of literature search.**

From the 27,897 entries identified from electronic resources, two interventional studies (Chlebowski et al. 2006; Djuric et al. 2002) (baseline n = 2,559; 2,259 analyzed) met our inclusion criteria (Figure 1). One trial was reported in two publications (Djuric et al. 2002; Chen et al. 2004).

Table 1 describes the characteristics of the included studies and their participants. Both studies were randomized trials conducted in the United States (Chlebowski et al. 2006; Djuric et al. 2002). The duration of the intervention was 12 months in both studies. One trial followed participants for an additional 48 months after the intervention (Chlebowski et al. 2006). The risk of bias was moderate for all studies due to lack of reporting on masking of the outcome assessors as to which intervention the participants were assigned to.

**Table 1:** **Characteristics of included studies**

Study	Cancer population	Study design	Stated goal of weight gain prevention	Baseline characteristics of participants		
Years of recruitment		Trial duration		Age	Race	Smoking status
Djuric et al. (2002)	At-risk for breast cancer	Single center randomized trial	No	Mean Age	%White	NR
1997-1999		12 months		Overall: 38 years	Overall: 75%	
Chlebowski et al. (2006)	Breast cancer	Multicenter randomized trial	No	Mean Age	%White	Current smokers, n
1994-2001		60 months		Overall: 58.6 years	Overall: 85.1%	Overall: 162

Abbreviations: *NR* Not reported.

### Prevention of weight gain in women with or at risk of breast cancer

One trial reported on an intervention to prevent weight gain in women at risk of cancer and another trial reported on an intervention among women with breast cancer (Chlebowski et al. 2006; Djuric et al. 2002). The trial of dietary interventions for prevention of weight gain in women at risk for cancer included premenopausal women (n = 160) with a family history of breast cancer and randomized these women to one of four diet groups for one year: control, low fat diet (LF), high fruits and vegetables diet (FV), or a combination of low fat and high fruits and vegetables diet (LFFV) (Table 2). As compared with the control group, the LF group lost weight. The FV and LFFV groups gained weight (Table 3). We were unable to calculate statistical significance for the between group differences based on the data reported. This study did not report on other outcomes of interest.

**Table 2:** **Description of included interventions**

Study	Intervention	Intervention arms			
		Control	Intervention Arm 1	Intervention arm 2	Intervention arm 3
			***LF Group***	***FV group***	***LFFV group***
Djuric et al. (2002)	Dietary	Received information on Food Guide Pyramid from National Dairy Council	Low fat diet (<15% calories from fat), one on one counseling with dietitians, monthly group meetings, written materials	High fruits/vegetables diet (≥9 servings daily), one-on-one counseling with dietitians, monthly group meetings, written materials	Combination of low fat and high fruits/vegetables diet, one on one counseling with dietitians, monthly group meetings, written materials
Chlebowski et al. (2006)	Self-management Dietary	Maintained usual diet. Had contact with dietician at baseline and every 3 months plus written materials	Low fat diet (<15% calories from fat) while maintaining nutritional adequacy. In person counseling sessions with focus on self-management strategies: biweekly plus dietician visits every 3 months	NA	NA

Abbreviations: *NA*, Not applicable.

**Table 3:** **Differences in weight change from baseline to 1 year measured in kilograms among women with cancer**

Study	Control	Intervention arms	Total number analyzed	Baseline weight (kg) control	Baseline weight (kg) Intervention arm	Mean change (kg) control group	Mean change (kg) intervention arm	Mean difference (kg) in group change
Djuric et al. (2002)	Diet/brochure	Low fat diet (LF)	49	66.3	70.3	−0.4	−5	−4.6
Djuric et al. (2002)	Diet/brochure	high fruits and vegetables diet (FV)	50	66.3	66.8	−0.4	1.8	2.2
Djuric et al. (2002)	Diet/brochure	Combo diet (LFFV)	48	66.3	68.9	−0.4	0	0.4
Chlebowski et al. (2002)	Dietary counseling	Low fat diet + Self management counseling	2164	72.6	72.7	0.2	−2.1	−2.3

Chlebowski et al. (2006) randomized women (n = 2,437) with breast cancer to a non-specific counseling group or an intervention group which received a combination of dietary counseling for a low fat diet with self-management techniques including goal setting, social support and dietary relapse prevention and management (Table 2). As compared with the non-specific counseling group, the low-fat group lost weight at one year. (Table 3) Similarly, BMI was 0.8 kg/m^2^ lower in the low fat group as compared with the non-specific counseling group at one year.

In this study, women who received low-fat dietary counseling had a 24% lower risk of breast cancer relapse and 29% lower risk of breast cancer recurrence than those who received non-specific counseling, after 5 years of follow up. There was no reported risk reduction in overall mortality. The study stated that no adverse events were associated with the dietary interventions.

## Discussion

Despite the rich literature on association of being overweight and obese to the risk of developing breast cancer (Ballard-Barbash et al. 2012; Rock et al. 2012; Norat et al. 2008; Norat et al. 2010; World Cancer Research Fund/American Institute for Cancer Research 2007) and to the poorer outcomes after the diagnosis (Ballard-Barbash et al. 2012); we identified only two studies, with strategies for weight maintenance, for inclusion in our review. Strategies that were effective included use of a low fat diet in premenopausal women with a family history of breast cancer; and group counseling on monitoring fat intake resulting in low fat diet in women with breast cancer. The present review highlights strategies that may effectively prevent weight gain among women with or at risk of breast cancer.

World Cancer Research Fund/American Institute for Cancer Research (WCRF/AICR) 2007 report (World Cancer Research Fund/American Institute for Cancer Research 2007) and American Cancer Society (ACS) guidelines on Nutrition and Physical Activity for Cancer Prevention (Kushi et al. 2012) recommended that individuals maintain a lean weight to prevent cancer. A body of original research also suggests that being overweight and obese increases the risk of postmenopausal breast cancer (Ewertz et al. 2011; Protani et al. 2010; Norat et al. 2008). Most of the data come from the cohort studies of breast cancer (Norat et al. 2008; Goodwin et al. 1999).

Studies have shown that breast cancer is diagnosed at early stages now and most of the patients are obese or over weight at the time of diagnosis (Pekmezi and Demark-Wahnefried 2011). It is further complicated by post- diagnosis weight gain secondary to several factors like side affects from the cancer treatment or lack of physical activity after the diagnosis (Chlebowski et al. 2002). The research lacks in the field of interventional studies to indicate how to achieve the goal of weight gain prevention at the different stages of cancer survivors i.e. at the time of diagnosis, during and post-treatment. Few interventional studies (Demark-Wahnefried et al. 2012) have reported the strategies of weight maintenance in individuals with breast cancer but were excluded from this review mainly due to lack of having one year follow up and lack of reporting on meaningful weight related outcomes.

The most extensive review published by WCRF/AICR (World Cancer Research Fund/American Institute for Cancer Research 2007) in 2007 recognized the lack of evidence about prevention of weight gain in cancer survivors. The ACS’s Nutrition and Physical Activity for Cancer Survivors (Rock et al. 2012) guidelines also acknowledged that most of the evidence related to weight maintenance or weight loss strategies does not come from studies of cancer survivors. Dietary and nutritional requirement are very different depending on the stage and type of cancer immediately post diagnosis. Thus ACS recommends maintaining healthy weight and consult with registered dietitian who is also certified specialist in oncology, achieve and maintain healthy weight, engage in regular exercise and consume diet high in vegetable, fruits and whole grains. The evidence to support these recommendations is derived from studies on general population and not cancer survivors. Research also lacks to show any strong benefit or risk in terms of mortality with adopting the weight maintenance strategies in early stages of cancer diagnosis in overweight or obese individuals.

### Limitations

Our review is limited by the paucity of the literature evaluating weight maintenance interventions in patients with or at risk for breast cancer. Also, one of the studies did not report variance estimates, so we were unable to calculate confidence intervals for our results to evaluate the statistical significance of the study results. Only one (Djuric et al. 2002) of the included studies had the stated goal of weight maintenance. While our results are suggestive for the women with breast cancer, we were unable to identify studies including other types of cancer, studies with men or individuals from different ethnic backgrounds.

## Conclusions

We identified two RCTs supporting strategies to prevent weight gain among women with or at risk of breast cancer, which included dietary counseling on low fat diet and weight reduction with low fat diet. Additional research is needed to confirm these strategies as effective in preventing weight gain and to assess whether weight gain prevention interventions also result in decreased cancer incidence, recurrence and mortality in other types of cancers and different populations.

### Consent

Our study population for a systematic review is medical publications. No individuals were directly contacted or studied as part of our work.

## Authors’ original submitted files for images

Below are the links to the authors’ original submitted files for images. Authors’ original file for figure 1
