# Supplementary material for: Comparative effectiveness of strategies to prevent weight gain among women with and at risk for breast cancer: a systematic review
Source: Springerplus. 2013 Jun 26;2(1):277. doi: 10.1186/2193-1801-2-277 (PMC3701789; doi:10.1186/2193-1801-2-277)
Supplement: Supplementary file 1 — Authors’ original file for figure 1 [file 40064_2013_353_MOESM1_ESM.pdf]

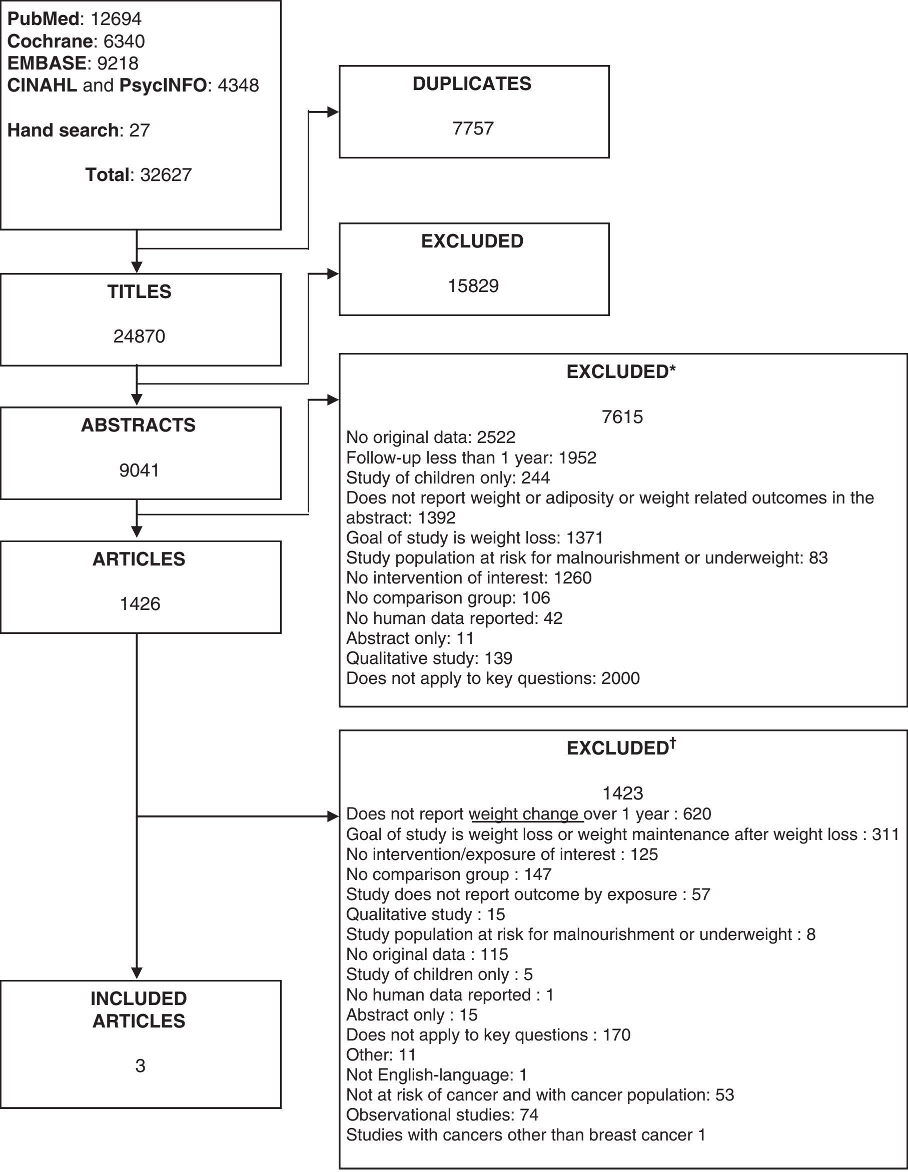

* Sum of excluded abstracts exceeds 7615 because reviewers were not required to agree on reasons for exclusion

† Sum of excluded articles exceeds 1421 because reviewers were not required to agree on reasons for exclusion
